# Expression of Nef from unintegrated HIV-1 DNA downregulates cell surface CXCR4 and CCR5 on T-lymphocytes

**DOI:** 10.1186/1742-4690-7-44

**Published:** 2010-05-13

**Authors:** Richard D Sloan, Daniel A Donahue, Björn D Kuhl, Tamara Bar-Magen, Mark A Wainberg

**Affiliations:** 1 McGill University AIDS Centre, Lady Davis Institute, Jewish General Hospital, Montréal, QC, Canada; 2 Department of Microbiology and Immunology, McGill University, Montréal, QC, Canada; 3 Department of Experimental Medicine, McGill University, Montréal, QC, Canada

## Abstract

**Background:**

Transcription of HIV-1 cDNA prior to, or in the absence of, integration leads to synthesis of all classes of viral RNA transcripts. Yet only a limited range of viral proteins, including Nef, are translated in this context. Nef expression from unintegrated HIV-1 DNA has been shown to reduce cell surface CD4 levels in T-cells. We wished to determine whether Nef expressed from unintegrated DNA was also able to downregulate the chemokine coreceptors CXCR4 and CCR5.

Viral integration was blocked through use of an inactive integrase or by using the integrase inhibitor raltegravir. Infected cells bearing unintegrated DNA were assayed by flow cytometry in the GFP reporter cell line, Rev-CEM, for cell surface levels of CD4, CXCR4 and CCR5.

**Results:**

In cells bearing only unintegrated HIV-1 DNA, we found that surface levels of CXCR4 were significantly reduced, while levels of CCR5 were also diminished, but not to the extent of CXCR4. We also confirmed the downregulation of CD4. Similar patterns of results were obtained with both integrase-deficient virus or with wild-type infections of cells treated with raltegravir. The Alu-HIV qPCR assay that we used for detection of proviral DNA did not detect any integrated viral DNA.

**Conclusions:**

Our results demonstrate that Nef can be expressed from unintegrated DNA at functionally relevant levels and suggest a role for Nef in downregulation of CXCR4 and CCR5. These findings may help to explain how downregulation of CXCR4, CCR5 and CD4 might restrict superinfection and/or prevent signal transduction involving HIV-1 infected cells.

## Background

Integration of the reverse transcribed HIV-1 genome into host cell chromatin is one of the defining features of retroviral replication and is mediated by the virally encoded integrase enzyme. During natural infections, unintegrated forms of HIV-1 cDNA can be detected in abundance *in vivo *[1-5] and in great excess relative to integrated DNA, despite normal integrase function [1,5]. Such unintegrated DNA can be found in three forms: linear cDNA that is the precursor to integrated proviral DNA, and 1- and 2-LTR circles that are the products of non-homologous end joining, autointegration, or recombination of linear cDNAs [6-8].

Although HIV-1 unintegrated DNA cannot itself support viral replication [9,10], it is transcriptionally active resulting in all classes of viral transcripts [8,11,12]. Translation of the early viral gene products such as Nef [13,14], Tat [10,15-17] and Rev [11] from viral mRNA of unintegrated DNA origin has been well documented; however, a key limitation in translation of late transcripts is low levels of Rev produced by unintegrated templates [11].

A detailed study of transcription using Rev-CEM cells, a CEM-SS derived cell line that had been transduced with a Rev and Tat dependent GFP expression vector [18], thereby allowing GFP analysis of infected cells [19], showed them to be permissive for transcription from unintegrated templates to approximately 70% of wild-type (wt) levels [20]. Earlier studies, using the Tat induced HeLa-CD4-LTR-β-galactosidase cell line, suggested that unintegrated transcription occurred to about 10% of wild type levels [16]. Other work identified a viral RNA transcript arising from across the LTR-LTR junction of 2-LTR circles [21], although its biological function, if any, remains undefined. Initial transcription from unintegrated DNA appears to be mediated by virally imported Vpr, as the presence of Vpr increased transcription from unintegrated DNA templates by 10-20 fold, and this process was found to be independent of Tat [8,22].

Although unintegrated DNA can be transcribed, it possesses no origin of replication and so is not maintained upon cell division. Therefore, the stability of unintegrated DNA in dividing cells is governed by the rate of cell division [23,24]. Insertion of an SV40 origin of replication into integrase-defective HIV-1 molecular clones or lentiviral vector genomes allowed the maintenance and transcription of unintegrated DNA in dividing cell populations [25,26]. It has also been shown that unintegrated DNA is stable in growth-arrested T-cells for 5-7 days [23,27,28]. Non-dividing macrophages were shown to contain unintegrated DNA for up to 21 days post infection, and transcription of a viral-borne luciferase reporter gene was detectable throughout [29]. Further work demonstrated that multiple unintegrated DNA forms were present in macrophages for up to 30 days post-infection, with viral RNA transcripts and Nef being detectable during this period in a manner that correlated with altered levels of cytokine expression [12].

Nef synthesized from unintegrated DNA has also been linked to the downregulation of cell surface CD4 in primary CD4^+ ^T-lymphocytes [14]. This was confirmed in the SupT1 cell line, in which cell surface CD4 downregulation by Nef of unintegrated DNA origin was shown to be dependent on Vpr-mediated Nef expression [8]. In other studies, pre-integration translation of Nef and Tat was shown to increase the activation state of resting T-lymphocytes, thereby rendering them more amenable to productive infection [13].

The expression of early gene products from unintegrated DNA seems to be a natural feature of the HIV-1 replication cycle [30,31]. In addition, the use of integrase strand transfer inhibitors (INSTIs), such as raltegravir, also leads to elevated levels of unintegrated HIV-1 DNA [32,33]. Unintegrated DNA derived from integration-competent virus blocked by INSTIs shows the same pattern of transcription as preintegrated virus or integrase-deficient virus [11].

When integration does occur, Nef-mediated downregulation of each of cell surface CD4 and the CXCR4 [34,35] and CCR5 [36] coreceptors has the benefit of restricting superinfection. This may protect the virus within the cell from cellular toxicities associated with superinfection, due to over-accumulation of unintegrated HIV genomes [37,38]. Additionally, downregulation of CD4, CXCR4 and CCR5 may reduce signaling via these receptors, which might otherwise trigger apoptosis, modulate viral transcription, and alter cellular chemotaxis in infected cells [39,40].

Downregulation of cell surface CD4 by Nef in primary CD4+ T-cells by unintegrated DNA is well established [8,14]. We now show that Nef derived from unintegrated DNA can also downregulate cell surface CXCR4 and CCR5.

## Results

### Nef is expressed from unintegrated DNA

**Figure 1:**
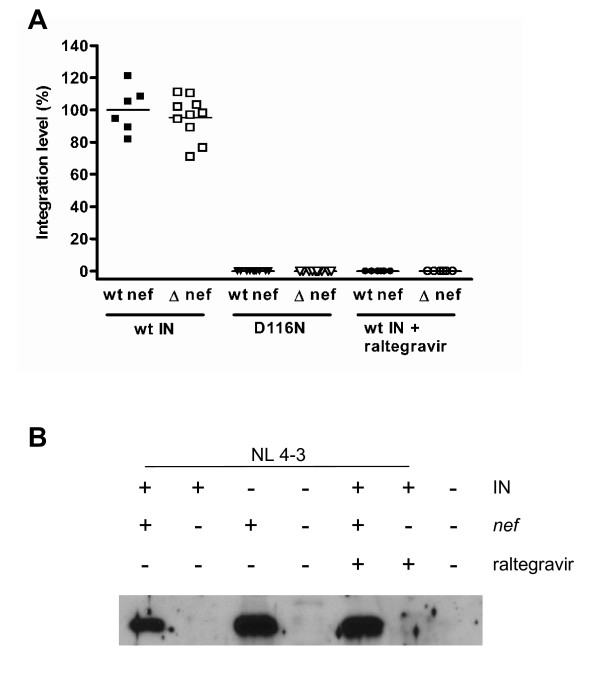
**Nef expression in the absence of integration**. **A**. Viral integration was measured by an Alu-HIV qPCR assay for provirus. Cells were infected with wild-type (wt) virus or D116N integrase-containing virus bearing either wt *nef *or Δ-*nef *mutations. Repeat infections were also performed for wt integrase virus in the presence of 1 μM raltegravir. At 72 h post-infection, DNA was extracted and qPCR analysis was performed. Results were expressed relative to those obtained with wt virus (levels of expression set at 100%). **B**. Expression of Nef was confirmed by Western blot analysis of lysates from infections with wt virus (IN +) or D116N integrase-containing virus (IN -), bearing either wt *nef *(*nef +*) or Δ-*nef *(*nef -*) mutations. Repeat infections were also performed for wt integrase virus in the presence of 1 μM raltegravir, a concentration shown to be completely inhibitory to integration.

We first sought to confirm that we could identify the expression of Nef in infections in which integration had not occurred [13]. Using an Alu-HIV qPCR for integrated provirus, levels of integration were expressed relative to those measured from infections using virus with a wild-type integrase at 72 h post infection. Neither infections with integrase deficient virus, bearing the D116N mutation, or wild-type integrase in the presence of 1 μM raltegravir, displayed measurable integration, i.e. the signal discernable from unintegrated cDNA was greater than that for the Alu-HIV amplification (Figure 1A).

Expression of Nef was analyzed by Western blot. In the absence of integration, i.e. infection with either integrase-deficient D116N virus or with wt virus in the presence of raltegravir, Nef expression still occurred at readily detectable levels (Figure 1B), thus confirming the translation of Nef from unintegrated DNA templates. Additionally, we confirmed that the introduction of two stop codons in the first three codons of the Nef gene was sufficient to prevent Nef synthesis

### Integrated virus downregulates cell surface CXCR4, CCR5 and CD4 expression on Rev-CEM cells

The Rev-CEM cell line was derived by transducing the Rev and Tat dependent GFP vector pNL-RRE(SA) [18] into CEM-SS cells, resulting in a CXCR4-and CCR5-bearing cell line that expresses GFP in response to the simultaneous presence of Tat and Rev [19]. Downregulation of CD4, CXCR4 and CCR5 by Nef is well established in the context of replication competent viruses [34-36]. In order to confirm that the Rev-CEM cell line was suitable for the study of Nef-mediated downregulation of cell surface receptors from cells bearing unintegrated viral DNA only, we first needed to confirm that Nef-mediated receptor downregulation was measurable following viral integration.

**Figure 2:**
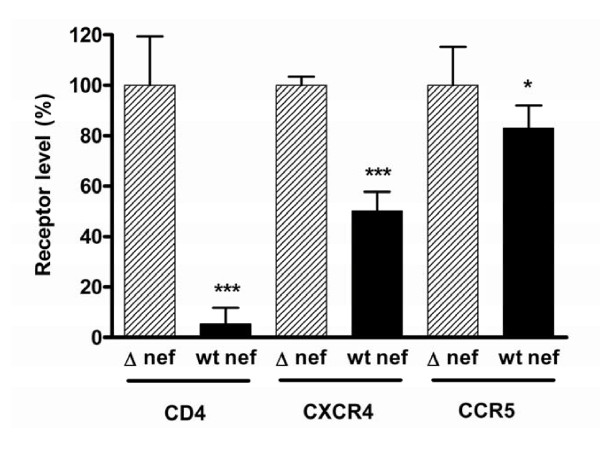
**Nef mediated downregulation of CXCR4, CCR5 and CD4 by integrating virus**. Infected (GFP positive) cells were analyzed relative to uninfected cells for cell surface CD4, CXCR4 and CCR5 after infection with wt integrase-containing virus, either wt *nef *or a Δ-*nef *mutation. The results of geometric means of fluorescence for each receptor are expressed relative to Δ-*nef *virus infection receptor levels. Results are from 3-5 independent experiments, each with two replicate infections. Error bars indicate standard deviations. For each receptor, statistical comparisons between wt *nef *and Δ-*nef *were performed by two-tailed unpaired *t*-tests, p < 0.001 (***) p < 0.05 (*).

Infected cells (i.e. GFP positive) were assayed by flow cytometry for cell surface expression of CD4, CXCR4 and CCR5 (Figure 2). Potent downregulation of CD4 by Nef was shown to occur, with cell surface levels being only ≈5% of those seen with Δ-*nef *viruses (p < 0.001) The CXCR4 coreceptor was also downregulated by Nef, to below 50% of levels attained with the Δ-*nef *virus (p < 0.001), whereas CCR5 downregulation was less, i.e. ≈83% of Δ-*nef *levels (p = 0.04).

### Integration deficient D116N virus downregulates cell surface CXCR4, CCR5 and CD4 expression

Having established the suitability of the Rev-CEM cell line to measure Nef-mediated downregulation, we next wished to study integrase-deficient virus, taking advantage of the capacity of unintegrated DNA to express Tat and Rev and thereby induce GFP expression [20]. Introduction of the D116N mutation into the integrase domain renders integrase inactive, and so cells infected with such virus will bear unintegrated viral DNA only [17]. Detection by Rev-CEM cells was sensitive for the detection of unintegrated infections by flow cytometry. With integrating virus, the infection rate inferred from GFP expression was typically 10%, and for integrase deficient virus typically 7% of the total population studied.

**Figure 3:**
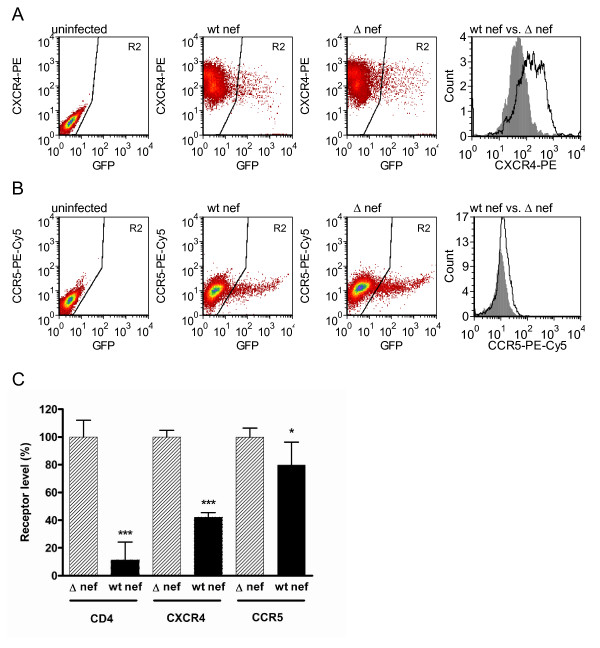
**Nef-mediated downregulation of cell surface CXCR4, CCR5 and CD4 by integrase-deficient (D116N) virus**. **A**. Flow cytometry dot plots demonstrating analysis of GFP positive cells in gate R2, depicting cells infected with integrase-deficient D116N virus. Cells infected with integrase deficient virus bearing the Δ-*nef *mutation demonstrate higher expression of CXCR4 than cells infected with wt *nef *virus. The histogram shows a direct comparison of CXCR4 levels for wt *nef *virus (shaded grey) and Δ-*nef *virus (black line, white background). **B**. Cells infected with integrase-deficient virus bearing the Δ-*nef *mutation demonstrate higher levels of expression of CCR5 than those infected by wt *nef *virus. The histogram shows a direct comparison of CCR5 levels after infection by wt *nef *virus (shaded grey) vs. Δ-*nef *virus (black line, white background). **C**. Cells infected with the integrase-deficient D116N virus were analyzed relative to uninfected cells for the presence of CD4, CXCR4 and CCR5 after infection with wt integrase virus containing either a wt *nef *or the Δ-*nef *mutation. The geometric means of fluorescence for each receptor are expressed relative to Δ-*nef *virus infection receptor levels. Results are from 3-5 independent experiments, each with two replicate infections. Error bars indicate standard deviations. For each receptor, statistical comparisons between wt *nef *and Δ-*nef *virus were performed by two-tailed unpaired *t*-tests, p < 0.001 (***), p < 0.05 (*).

Infected cells were measured by flow cytometry for cell surface expression of CD4, CXCR4 and CCR5. A pattern of downregulation, similar to that of integrating virus was observed. These findings confirm that Nef derived from unintegrated HIV-1 DNA can downregulate cell surface CD4 to levels ≈ 11% of those attained with Δ-*nef *virus (p < 0.001) (Figure 3). As the data were normalized to internal controls, direct comparisons between integrating vs. non-integrating viruses were not made.

We have also shown that Nef expressed from unintegrated DNA also diminished levels of expression of CXCR4 to ≈ 42% of those attained with Δ-*nef *virus, (p < 0.001). In contrast, downregulation of CCR5 in the same system only occurred to a level of ≈ 80% of that seen with the Δ-*nef *virus (p < 0.02).

### Integration competent virus downregulates cell surface CXCR4, CCR5 and CD4 expression in the presence of inhibitory concentrations of raltegravir

Having established that integrase-deficient virus could express Nef and downregulate levels of expression of entry receptors (Figure 3), we next wished to establish whether such down-modulation would also occur in the presence of an INSTI such as raltegravir. Previous work had established that 1 μM of raltegravir was sufficient to prevent measurable integration in the Rev-CEM cell line by qPCR for proviral DNA (Figure 1A). We therefore performed a series of infections with wt *nef *and Δ-*nef *virus to determine patterns of receptor downregulation in the presence of raltegravir.

**Figure 4:**
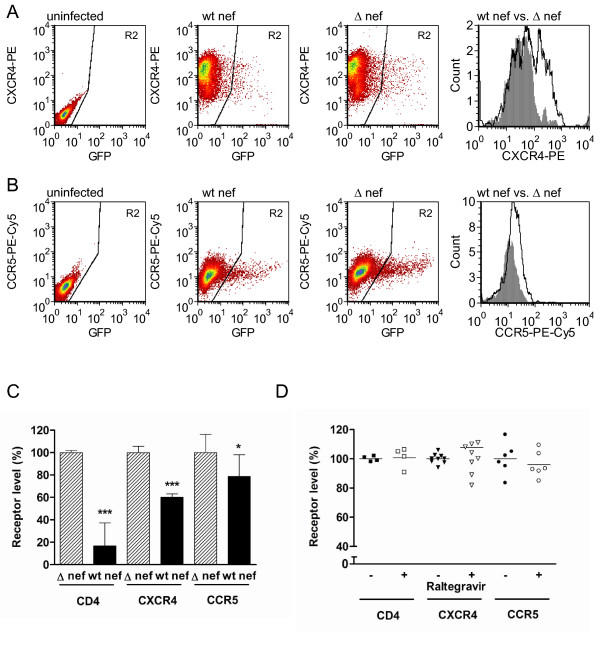
**Nef-mediated downregulation of cell surface CXCR4, CCR5 and CD4 by IV-1 infection in the presence of raltegravir**. **A**. FACs plots demonstrating analysis of cells infected with wt integrase virus in the presence of 1 μM raltegravir. GFP-positive (infected) cells are depicted in gate R2. Cells infected with Δ-*nef *virus demonstrate higher expression of CXCR4 than occurs for wt *nef *virus. The histogram shows a direct comparison of CXCR4 levels for wt *nef *virus (shaded grey) vs. Δ-*nef *virus (black line, white background). **B**. Cells infected with Δ-*nef *virus in the presence of 1 μM raltegravir demonstrate higher-level expression of CCR5 than cells infected by wt *nef *virus. The histogram shows a direct comparison of CCR5 levels for wild-type *nef *virus (shaded grey) vs. Δ-*nef *virus (black line, white background). **C**. Cells infected with wt virus containing either a wt *nef *or the Δ-*nef *mutation in the presence of raltegravir were analyzed relative to uninfected cells for the presence of cell surface CD4, CXCR4 and CCR5. Geometric means of fluorescence for each receptor are expressed relative to infection by the Δ-*nef *virus. Results are from 3-5 independent experiments, each performed in duplicate. Error bars indicate standard deviations. For each receptor, statistical comparisons between wt *nef *and Δ-*nef *viruses were made by two-tailed unpaired *t*-tests, p < 0.001 (***), p < 0.05 (*). **D**. 1 μM raltegravir does not directly influence cell surface levels of CD4, CXCR4 and CCR5. Uninfected cells were treated in the presence or absence of 1 μM raltegravir and then stained for CD4, CXCR4 or CCR4. Plots display expression levels relative to untreated cells. Results are from 3-5 independent experiments, each performed in duplicate. For each receptor, statistical comparisons between untreated and treated cells were made by two-tailed unpaired *t*-tests. No statistically significant differences were found.

Similar results to those for integrase-deficient virus were obtained (Figure 4), with cell surface levels of CD4 being reduced to 17% of levels attained with wild-type Δ-*nef *virus (p < 0.001). CXCR4 and CCR5 levels were reduced to 60% and 79% of those attained with Δ-Nef virus (p < 0.001 and p = 0.03, respectively). Direct comparisons between integrase-deficient and integrase competent viruses in the pressure of raltegravir were not made, as the experiment was internally controlled.

Finally, the results of Figure 4 show that there was no direct effect of raltegravir on expression of any of CD4, CXCR4 or CCR5 in this system.

## Discussion

We herein provide the first evidence of chemokine coreceptor downregulation mediated by Nef derived from unintegrated DNA. In addition, we confirm the findings of other groups that Nef expressed from unintegrated DNA can downregulate cell surface CD4 [8,14]. It may not be possible to make direct comparisons between our and other studies, due to different methods of flow cytometry employed.

In our studies, levels of downregulation of Nef-mediated CXCR4 derived from unintegrated DNA correlated well with results obtained in productive infection and are also in agreement with the finding that such downregulation occurs to a lesser extent than is seen for CD4 [34,35]. Although we observed a slightly lesser degree of downregulation of CCR5 by Nef from unintegrated DNA than has been reported for productive infection of activated primary human peripheral blood lymphocytes, our results are broadly consistent with the ≈ 25% downregulation of CCR5 seen with integrated infections of TZM/bl cells [36]. Studies with 293-Affinofile cells, a cell line that is quantitatively inducible for both CD4 and CCR5 cell surface expression, revealed that even modest downregulation of CCR5 from the cell surface was sufficient to impact infectibility, particularly in the context of reduced CD4 levels [41-43]. In our system as well, the levels of downregulation of CCR5 and CD4 that we report can probably limit viral entry.

With our methodology, one would not expect that Env would contribute to receptor downregulation, as we used *env *deleted pseudovirus bearing a VSV-G envelope. Additionally, although Vpu can act to downregulate CD4, there is currently no evidence that Vpu can modulate levels of CXCR4 and CCR5. Further, Vpu is not synthesized from unintegrated cDNA, and is therefore unlikely to affect cell surface CD4 levels.

An important consideration is that patterns of transcription and translation from unintegrated virus, arising from D116N integrase mutations or INSTI-treated cells, are identical to those observed in infections of quiescent T-cells prior to integration [13]. Further, there are insufficient levels of 2-LTR circles in integrase-deficient infections of Rev-CEM cells to account for the numbers of transcriptionally active cells, the inference being that unintegrated linear cDNA molecules, rather than 2-LTR circles, are the likely template for transcription [20]. Thus, blockage of integration can be informative in regard to transcription from linear cDNAs. Slowly replicating cells such as resting T-cells [13,44] and non-replicating cells such as macrophages [12] display a lag in transcription prior to integration. Cells in this state comprise the pre-integration latent reservoir [45]; and transcription during this period may be beneficial in regard to restricting superinfection, that may in turn be deleterious for cell viability and hence the likelihood of productive infection [37,38]. Rev can regulate integration [46] and, in addition, Rev generated from unintegrated DNA can act to restrict superinfection [47]. Downregulation of entry receptors may provide similar benefit, as is also seen with integrating infections [34,36]. Of course, recombination between unintegrated DNA and superinfecting virus might still occur as has been observed *in vitro *[48,49].

Downregulation of coreceptors by unintegrated DNA may also reduce cell-signaling due to stimulation by natural ligands or viral envelope. This may help to avert adverse effects such as chemotaxis, apoptosis, and changes in viral transcription [50-54]. Further, there may be an immunological benefit for Nef-mediated downregulation of MHC-I by unintegrated DNA, which may result in evasion from cytotoxic T-cell mediated lysis [55].

In summary, we have provided further evidence that Nef translation from unintegrated DNA can occur at functionally relevant levels, and leads to reduced cell surface expression of CXCR4 and CCR5 as well as CD4. Additional work to determine the benefits of coreceptor downregulation for virus-infected cells is now in progress.

## Methods

### Plasmids and cloning

The HIV-1 molecular clone pNL4-3 was altered through site-directed mutagenesis (Stratagene) to introduce termination codons in the first and third amino acids of the *env *gene (construct termed pNL4-3-ΔE). Further modifications by mutagenesis included the substitution D116N in the integrase coding sequence of the *pol *gene (construct termed pNL4-3-ΔE-D116N) and the introduction of termination codons into the first and third codons of the *nef *gene (constructs termed pNL4-3-ΔE-ΔN and pNL4-3-D116N-ΔE-ΔN).

### Virus production

Pseudovirus was produced by cotransfection via lipofectamine (Invitrogen) of 7 × 10^6 ^293T cells with 4 μg pVPack-VSV-G (Stratagene), a vesicular stomatitis virus G protein (VSV-G) envelope-encoding construct, in combination with 12 μg of a pNL4-3 derivative (either pNL4-3-ΔE, pNL4-3-D116N ΔE, pNL4-3-ΔE-ΔN or pNL4-3-D116N-ΔE-ΔN).

All transfection supernatants were harvested at 72 h post transfection, clarified by centrifugation for 5 min at 470 g, and passed through a 0.45 μm filter. Virus was treated with 50 U/ml benzonase at 37°C for 20 minutes to digest contaminating plasmid DNA [56] and then stored at -80°C until use.

### Cell culture and viral infections

CXCR4-and CCR5 bearing Rev-CEM cells [19] were obtained through the NIH AIDS Research and Reference Reagent Program (courtesy of Professor Yuntao Wu) and were maintained in RPMI 1640 medium (Invitrogen), and 293T cells were maintained in DMEM (Invitrogen), each supplemented with 10% fetal bovine serum, 1% L-glutamine and 1% penicillin/streptomycin.

1.25 × 10^5 ^Rev-CEM cells were infected with 500 ng p24 of virus in 24 well plates by spinoculation at 1200 g at 25°C for 2 h followed by 2 h at 37°C, after which medium was replaced, resulting in a multiplicity of infection (MOI) of 0.1 for wt virus as determined by GFP expression. Cells were infected with wt *nef *or Δ-*nef *virus that was either integrase competent (wt) or that contained a defective D116N mutated integrase. Additional infections were performed with wt integrase-containing pseudoviruses. In some cases, media were pre-treated with 1 μM final concentration raltegravir (a gift from Merck Canada, Inc) for 1 h prior to infection; after spinoculation, raltegravir-containing media were again used at a concentration of 1 μM.

### Integrated DNA qPCR

For the integrated DNA qPCR assays, cellular DNA was extracted with a DNeasy blood and tissue kit (Qiagen). PCR was performed with Platinum qPCR SuperMix-UDG (Invitrogen) on a Corbett Rotor-Gene 6000 thermocycler.

A previously described Alu-gag PCR analysis was used [57] with the following modifications [58]. The first round reaction was performed on undiluted samples (100 ng template) and 1:10 dilutions of each sample (10 ng template diluted with uninfected DNA; 100 ng DNA total) in the presence of 2 mM MgCl_2 _and 200 μM dNTPs. 9 μl of the resulting first round product were used as template for the second round nested reaction in the presence of 5 mM MgCl_2 _(final concentration including MgCl_2 _carryover from first round) and 200 μM dNTPs, using the "wild-type" probe only. Second round cycling conditions were 50°C for 2 min, 95°C for 1 min, and 45 cycles of 95°C for 15 sec and 60°C for 30 sec. Dual-labeled probes were obtained from Biosearch Technologies (Novato, CA, USA). To generate a standard curve for relative quantification of integrated DNA, Alu-gag PCR was first performed on a dilution series of DNA from infected Rev-CEM cells (diluted with DNA from uninfected cells).

### Western Blot

2 × 10^5 ^Rev-CEM cells were infected with pNL4-3-ΔE, pNL4-3-D116N ΔE, pNL4-3-ΔE-ΔN or pNL4-3-D116N-ΔE-ΔN, in the presence or absence of raltegravir. The cells were collected after 72 hours and pelleted by low speed centrifugation at 470 g. The pellet was resuspended in RIPA buffer (0.15 M NaCl, 20 mM Tris pH 7.4, 2 mM EDTA, 1% Triton X-100 and 1% deoxycholate). Cell lysates were normalized by Bradford assay to 1 mg/ml total protein and resolved in a 12% SDS-PAGE gel. The blot was incubated for 60 minutes with 1:4000 polyclonal anti-HIV-1 Nef antibody obtained from the NIH AIDS Research and Reference Reagent Program (catalog number 2949) and anti-rabbit IgG AP conjugate (secondary antibody) (1:10,000). A chemoluminescent reagent West-Pico (Pierce) was used to develop the blots.

### Cell surface CXCR4, CCR5 and CD4 staining

Rev-CEM cells that had been infected with pseudovirus were stained at 72 h post-infection in PBS containing 3% fetal bovine serum and 0.05% sodium azide for 30 minutes at 4°C with the following mouse monoclonal antibodies (MAbs): allophycocyanin (APC)-conjugated anti-human CD4 (clone RPA-T4; BD PharMingen); phycoerythrin (PE)-conjugated anti-human CXCR4 MAb (clone 12G5; BD PharMingen); PE-Cy5-conjugated anti-human CCR5 MAb (clone 2D7 BD PharMingen). Cells were then fixed in a final concentration of 1% paraformaldehyde, and then resuspended in PBS containing 3% fetal bovine serum and 0.05% sodium azide. 10,000 events were assayed on a FACSCalibur instrument (BD PharMingen); analysis was performed with BD CellQuest Pro 4.0.2 (BD PharMingen) and FCS Express 3 software (DeNovo). Levels of receptors were quantified relative to those found after infection by Δ-*nef *virus. These studies were controlled for by subtracting background isotype fluorescence values from antibody-receptor fluorescence measurements.

### Statistical analysis

All statistical analyses were performed with GraphPad Prism 4.0 software. To test for statistically significant differences between groups, unpaired two-tailed *t*-tests were performed with confidence intervals set at 95%.

## Abbreviations

INSTI: Integrase strand transfer inhibitor.

## Competing interests

The authors declare that they have no competing interests.

## Authors' contributions

RDS designed the study, performed the experiments and drafted the manuscript. DAD helped design the study and performed qPCR optimization. BDK helped with plasmid construction and flow cytometry analysis. TB-M performed Western blots and assisted in the study design. MAW provided overall supervision for the project, secured funding, and helped write the manuscript. All authors read and approved the final manuscript.
